# Implications of the COVID-19 Pandemic on the Family Structural Dimensions: A Correlational Study

**DOI:** 10.3390/ejihpe13090115

**Published:** 2023-08-25

**Authors:** Margarida Silva, Manuela Ferreira, Helena Loureiro, Teresa Kraus, Angelica Santos, Maria Henriqueta Figueiredo

**Affiliations:** 1 Nursing Health Sciences Research Unit, Nursing School of Coimbra, Avenida Bissaya Barreto, 3046-851 Coimbra, Portugal; 2 Nursing School of Oliveira de Azeméis, CINTESIS@RISE Rua da Cruz Vermelha, 1002, 3720-126 Oliveira de Azeméis, Portugal; manuela.ferreira@essnortecvp.pt; 3 Health School, University of Aveiro, 3810-193 Aveiro, Portugal; hloureiro@ua.pt; 4 Health School, Polytechnic Institute of Leiria, Morro do Lena, Alto do Vieiro.4137, 2411-901 Leiria, Portugal; teresa.kraus@ipleiria.pt; 5 Family Health Unit, Grouping of Health Centers Póvoa de Varzim/Vila do Conde, 4485-460 Vila do Conde, Portugal; aafsantos@arsnorte.min-saude.pt; 6 Nursing School of Porto, CINTESIS@RISE, Rua Dr. António Bernardino de Almeida, 4200-072 Porto, Portugal; henriqueta@esenf.pt

**Keywords:** COVID-19, pandemics, family nursing, family characteristics, family structure, work–life balance

## Abstract

The COVID-19 pandemic caused changes in the families’ social support network, employment status, and family income, which are the focus of attention of family health nurses. This study aims to describe the pandemic’s repercussions in the areas of attention of the structural dimension of families according to the Dynamic Model of Family Assessment and Intervention, as perceived by Portuguese families, and to relate the changes in their employment status according to the variables of the structural dimension. A quantitative, descriptive, and correlational study was conducted using snowball sampling. A questionnaire of sociodemographic characterization and assessment of the family structural dimension according to the model’s operational matrix was applied to 235 family members. Changes were found in their employment status; family income; intensity and frequency of contact with family, friends, and coworkers; frequency of contact with educational/health/religious institutions and community groups; cultural activities; and housing comfort conditions such as the use of heating/air conditioning, gas, and water consumption. Changes in employment status were related to family income, interaction with friends, frequency of cultural activities, and use of air conditioning and heating. Knowing the implications of the pandemic on the family’s structural dimension results in a nursing intervention more focused on family needs.

## 1. Introduction

The lockdown and social isolation resulting from public health measures during the COVID-19 pandemic have caused macro-structural changes to families’ economic, social, and labor status [1], impacting their dynamics [2,3].

The financial, job, and housing insecurity and the deprivation of support from friends and family resulting from the pandemic have caused harm to the physical and mental health of family members, which, in turn, manifests in altered family interaction and patterns [2,4,5]. Fear and uncertainty, in addition to stress from the restrictions and constraints of daily life, have particularly affected low-income families, families from ethnic minorities, and vulnerable groups and women [6,7]. 

For stable family dynamics in the face of a new aggravated job, financial insecurity, and social activity situations, families were forced to adapt, particularly at the structural level, to meet their needs by identifying and enhancing their intrinsic strengths [8]. However, families’ response to the employment and economic changes resulting from the pandemic in 2020 occurred differently, negatively impacting family relationships [7]. According to the same authors, these effects on family relationships depended mainly on the quality of relationships and family well-being before COVID-19. 

Long et al. [9] identified four domains affected by the pandemic and the consequent public health response that contributed to drastically altering social interactions: social networks, social support, social interaction, and intimacy. These four domains integrate the operative matrix of Figueiredo’s Dynamic Model of Family Assessment and Intervention (MDAIF) [10] regarding the frequency of contact between the household and extended family and the specification of the roles of the extended family network (social companionship, emotional support, cognitive guidance and advice, social regulation, material help and services, and access to new contacts). 

The MDAIF has three evaluative dimensions: the structural, developmental, and functional dimensions [10]. The structural dimension integrates the following focal areas: the family composition (identification of family members, and birth order and subsystems—family dynamic and ecological assessment, family type, family social class, and family income), the existing links and other subsystems between the family and the extended family (type and frequency of contact and roles of the relationships: social companionship, emotional support, cognitive guidance and advice, social regulation, material help and services, and access to new contacts) and the broader systems, that is, social institutions or significant persons not belonging to the extended family and, also, specific environmental aspects (family income residential building, safety precautions, housing hygiene, existence of architectural barriers and safety conditions, water supply system, and biological environment) that may constitute a health risk.

According to the MDAIF [10,11], nursing care focuses on promoting family health projects, using internal and external resources to endorse new forms of interaction that strengthen the family’s health and autonomy, where nurses facilitate the co-construction of solutions. 

The MDAIF [10] also allows for characterizing the family system, identifying relational and role patterns associated with the development and non-normative transitions resulting from other internal or external fluctuations that generate family crises, such as the COVID-19 pandemic.

As the pivots of the interaction with families, primary health nurses are the most skilled and competent health professionals to develop interventions that promote the ability to identify needs and mobilize resources for their adapted functioning [10,12]. 

The objectives of this study are as follows:

- To describe the implications of the COVID-19 pandemic in the focus areas of the MDAIF structural dimension, as perceived by Portuguese families;
- To relate the changes in the employment status perceived by families according to the variables of the MDAIF structural dimension.

## 2. Materials and Methods

### 2.1. Research Design

A quantitative, descriptive, and correlational study was carried out. The objective was to assess the effect of the COVID-19 pandemic on family dynamics, considering the focus areas described in the structural dimension that integrate the following operating matrix of the MDAIF [10]: employment status; family income; intensity and frequency of contact with family, friends, and coworkers; frequency of contact with educational/health/religious institutions and community groups; cultural activities; and housing comfort conditions such as the use of heating/air conditioning, gas, and water consumption.

### 2.2. Participating

The non-probability sample, composed of 234 families, was obtained from the personal social network of the project researchers, using the snowball technique, in which participants invited others, and so on. Inclusion criteria were families living in Portugal willing to participate in this study. 

### 2.3. Procedures

Data were collected between January and June 2021.

Anonymity and data confidentiality were guaranteed, as well as individual consent to participate in this study and other ethical principles. The data file was encrypted and destroyed at end of this study. This study was submitted to the Ethics Committee of the Health Sciences Research Unit: Nursing (UICISA: E) of the Nursing School of Coimbra (ESEnfC) and obtained a favorable opinion (Opinion No. P732-11/2020).

### 2.4. Instruments

The data collection instrument was self-completed online, using Google Forms, and consisted of two parts. The first part comprised a family characterization questionnaire (type of family, composition, existence of a family member with COVID-19 or in prophylactic isolation). The second part included questions that evaluate the effect of the COVID-19 pandemic on the family’s structural dimension, based on the care areas of the MDAIF [10], which can be scored on a Likert-type scale from 0 (nothing changed) to 7 (everything changed). In cases where the participants registered the change with a score higher than 4, the direction of this change (increased/decreased) was questioned using a dichotomous scale.

### 2.5. Data Analysis

Data were processed using the IBM^®^ SPSS Statistics V27 program. 

For the descriptive analysis of the data, measures of central tendency and dispersion and absolute and relative frequencies were used. The Kolmogorov–Smirnov test was performed to assess the nature of the sample distribution. Inferential statistical analysis was conducted with the non-parametric Kruskal–Wallis test.

## 3. Results

Of the 234 participants, approximately 60% belong to nuclear families (59.8%), followed by married couple families (12.4%), single parent families (9.8%), single person families (7.7%), reconstructed families (4.7%), extended families (3.8%) and others (1.7%).

Participants’ households averaged 3.31 (±1.17) members, ranging from one to six members, and 73.1% of participants identified the presence of the marital subsystem in their households.

Approximately half of the participants (n = 115; 49.1%) reported changes in their employment status due to the COVID-19 pandemic. Of the changes in their employment status, 59.6% started working as teleworkers, 10.4% were on furlough, 6.9% started a labor or service contract, 5.2% became unemployed, and 2.6% retired.

Of all the participants, 16.7% reported the existence of a person with COVID-19 in the family, and 40.2% reported having at least one family member in prophylactic isolation.

Table 1 shows the results of the changes in the focus areas of family income, safety precautions, and water supply in the family structural dimension according to the MDAIF [10].

The family income of the families showed a low degree of change (mean of 2.51 ± 1.70). Of the families that presented changes with a value greater than 4, 67.1% reported decreasing family income, while 32.9% reported increasing family income.

Regarding the intensity of contact with extended family members, families showed a mean change of 5.29 ± 1.92, with 96.3% reporting a change greater than 4, reflecting a decrease in contact intensity.

Regarding the support given to the household by the extended family members, an average of 3.71 ± 2.14 was found, with a decrease of 78% in social companionship, 64.8% in emotional support, and 69.6% in material help and services.

The interaction with coworkers showed a mean change of 4.71 ± 1.91, with a decrease in 88.3% of the participants who registered changes with an intensity of 4 or more.

The interaction with friends showed a mean change of 5.75 ± 1.56, and 99% of those who reported change equal to or greater than 4 registered a decrease in interactions.

Regarding interaction with health, educational, and religious institutions or community groups, the mean changes were 3.97 ± 2.06; 4.53 ± 2.22; 3.71 ± 2.48; 4.18 ± 2.45, respectively. The interactions with health, educational, and religious institutions or community groups, with an intensity value of 4 or more, showed a decrease in contact of 92.9%; 89.5%; 96.5%, and 98.5%, respectively.

As for leisure and cultural activities, participants showed high mean changes of 5.74 ± 1.58 and 6.04 ± 1.54, respectively, reflected in decreases of 98 and 99%, respectively, in participants reporting changes of level 4 or higher.

With regard to the use of heating/air conditioning, gas, and water consumption, the mean change was 3.77 ± 2.24, 3.28 ± 2.27, and 4.14 ± 2.09, respectively. All participants who recorded a change of 4 or more on the Likert-type scale reported an increase in the consumption of these resources, with a 96% increase in the use of heating/air conditioning, a 92.5% increase in gas consumption, and a 98.6% increase in water consumption.

Table 2 shows that the variable “what changed in the employment status” did not show statistically significant correlations with the variables “intensity of contact with extended family”, “support from extended family”, “interaction with the social network (coworkers, friends, health institutions, educational institutions, and community groups)”, and “water consumption”.

The variable “what changed in the employment status” showed a correlation with the variable “employment status” (*t* = 13.913; *p* = 0.031), meaning that those unemployed, who started a work contract, and who were on furlough perceived greater changes in their employment status. Family income registered more significant changes (*t =* 24.032; *p* = 0.001) among those unemployed, retired, and who started a work contract. The frequency of cultural activities changed significantly (*t* = 14.217; *p* = 0.027) in participants who started telework, had a service contract, and became unemployed. 

The use of heating/air conditioning was significantly influenced (*t* = 12.824; *p* = 0.046) by starting work and telework. Also, gas consumption underwent statistically significant changes (*t* = 13.341, *p* = 0.038), especially in participants who were on furlough and teleworkers. 

## 4. Discussion

The results highlighted the readjustment of Portuguese families during the COVID-19 pandemic. Theoretically supported via the MDAIF, the impact of this phenomenon was particularly evident in the structural dimension due to the changes it caused in most of the participating family systems. 

Families perceived a change, with a low mean value, in their family income. This evidence is corroborated by Camarano [2], Lebow [13], and Wang et al. [5], who stated that the important implications in family dynamics derive from changes in employment status, namely unemployment, often a consequence of the pandemic. Also, Correia [14] concluded that the COVID-19 pandemic would leave an indelible mark on individuals and families in future years, particularly in families with lower economic resources.

A change is observed in contact with families, with a decrease in its intensity. This study’s results corroborate Lebow [13] and Long et al. [9], which pointed to a reduction in the intensity of contact within the household, with a loss of personal relationships and intimacy. Contrary to these sources and to what could be expected due to the global protection measures, this study found, although in a small percentage, an increase in face-to-face contact related to the concern of families for their older members, although mostly non-face-to-face. A study conducted in Estonia [15] concluded that family environments proved positive during the first wave of the pandemic in 2020, which could alleviate the harmful effects of confinement. Another study conducted in Spain [16] found that confinement allowed families to enjoy quality time with their members, promoting opportunities for fun and relaxation, thus highlighting the importance of family leisure in reducing anxiety levels. However, a study from Estonia [15] found that children showed higher levels of fatigue and boredom during the second outbreak of the pandemic in 2021, impacting family relationships. 

The extended family’s decreased support to the household, expressed by decreased social companionship, emotional support, and material help and services, highlighted the fear response that plagued families at the time. Contingency measures restricting physical interaction between households carry heavy and unequal relational costs (notably for those living alone, differences in age groups, religious/ethnic groups, and social classes) [9]. Increasing evidence [9] suggests that online forms of relationships, while very useful, are no substitute for physical interactions. 

This study also found changes in all social interactions, resulting in a decrease in the frequency of contact. In descending order of frequency, this change occurred in the interaction with friends, coworkers, educational institutions, community groups, health institutions, and finally, with less change, religious institutions. Also, this study found a significant reduction in the frequency of cultural and leisure activities. In addition to health policies to contain the spread of the virus, this finding illustrates the extreme isolation of Portuguese families against the possibility of contracting the SARS-CoV-2 disease (COVID-19) at a time when treatment and prognosis were guarded. 

Interaction with coworkers and friends had a decrease in physical contact, as was observed with the extended family. The very decision of COVID-19 contingency plans in making telework mandatory, in addition to the unequal digital access and digital literacy provided by family differentiation, had an impact on “group properties” and the well-being of workers with a consequential impact on their family dynamics [9]. The authors also emphasize the intelligent combination of online and face-to-face work and more equity in digital literacy among workers to reduce the inequity of opportunities at this level [9]. The analysis of the first and second waves of the COVID-19 pandemic in Estonia in 2020 and 2021, respectively, found an increase and change in the quality of online contact time of children, a situation that did not compensate for the loss of real-life interaction with friends [15].

Regarding the interaction with institutions, the heterogeneity of response may have been due to the meaning given to them. The study by Long et al. [9] identified the impact of the COVID-19 pandemic restrictions on people and the local economy and suggested the creation of relational benefits and stronger relationships near their homes or residential areas. In this context, Long et al. [9] introduce the concept of the “support bubble” as a systemic, sustainability-based intervention between local governance, commerce, and community groups to build more robust and sustainable localized communities in a mix of offline and online interactions, creating opportunities to capitalize on the potential of more localized communities in response to pandemics.

The results showed little change in the demand for health and religious institutions and community groups as a source of support, probably because they were closed or with restricted access, or even due to the lack of need to seek them, for prioritization reasons associated with the fear of contagion.

The increase in the use of heating/air conditioning, gas, and water consumption was related to the greater permanence of families at home, a result of policy measures to mitigate social contact, and water consumption was, of all consumption, the one with the most significant increase. These results corroborate those of a study conducted in Canada by Rouleau and Gosselin [17], where general consumption (electricity, hot water, and space heating) increased slightly, with the higher consumption occurring throughout the day instead of at night as observed before the pandemic confinement.

Several changes were perceived by families in their employment status, according to the variables of the MDAIF structural dimension [10], which several authors have corroborated. A study by Chen, Byrne, and Vélez [18] concluded that short-term work was associated with the perception of a negative impact on professional life, which corroborates, in part, the results of this study. In contrast, telework was strongly associated with a perceived positive impact on professional life. 

Unemployed individuals, those worried about SARS-CoV-2 contamination, and people from families with a greater focus on emotion, problem, or support were the most likely to increase artistic and cultural involvement. Indeed, artistic activities were used as emotion management strategies, contributing to improved self-development [19].

However, some authors warn that prolonged confinement diminished the regularity of social connections in copresence and physical proximity and increased socioeconomic vulnerability, leading to disruptive processes in the later period that may cause changes in the very notion of society [19]. To minimize the impact of poverty and social isolation in Portugal, the energy supply regulator determined that households could pay their electricity, gas, and water debts in installments and interest-free [14]. Some studies [20] have also evidenced that lower-income or higher-income households experienced the largest increases in energy and air conditioning consumption, while middle-income households experienced more minor changes. These findings reinforced the social imbalances and their consequences experienced during the pandemic, mainly resulting from unemployment or adaptation to telework, influencing the social conditions of individuals and their families, including income, housing conditions, education, and gender, among others [19].

The main limitation of this study is the sampling process that, not being randomized and representative, does not allow extrapolation of the results to the Portuguese population. On the other hand, the fact that data were collected via an online questionnaire may also have limited the heterogeneity of the sample and the high detection of non-response in its full completion.

The results of this study highlight the need for further studies on the assessment of the family’s structural dimension and on more adequate nursing interventions that meet the family’s needs in crises.

Despite the dramatic changes in social interaction during a pandemic, significant challenges and opportunities for development were created in family health. It is essential to learn how to attribute meaning coherently to the challenges [18], in this case, to the implications of the COVID-19 pandemic in family dynamics and also the need to choose firm values, a liberating attitude, promoting resilience and hope, essential virtues to overcome anguish. In this sense, the evidence shows the need for responsive therapeutic nursing practices that guide toward family empowerment and cohesion [21]. The pandemic informs new research directions, clinical approaches, and policy issues at the individual, community, and societal levels [22].

## 5. Conclusions

This study identified the changes in the structural dimension according to the MDAIF perceived by Portuguese families during the COVID-19 pandemic. Changes occurred in the employment status, family income, intensity and frequency of contact with family, friends, and coworkers, frequency of contact with educational/health/religious institutions and community groups, cultural activities, and use of heating/air conditioning, gas consumption, and water consumption. The changes in the employment status resulting from the pandemic were related to family income, socializing with friends, frequency of cultural activities, and the use of air conditioning and heating. 

The results of this study show that systems to nursing record systems should include the assessment of the family structural dimension so that the nurses’ intervention focuses more on the family’s needs and strengths, particularly during pandemics. Teleconsultation, as an alternative or complement to face-to-face consultation, should be a standard practice to increase the accessibility of families to health care, thus being able to provide cognitive guidance, support, and material help and services, that reflect on the family’s health and well-being.

Healthcare teams should predict unequal impacts on the areas of attention of the MDAIF structural dimension in care planning to families in future pandemics or other crises. 

This study also makes it possible to reflect on the responses of the health sector and other sectors to the needs of families in the face of changes in the variables of the structural dimension caused by the pandemic or another non-normative crisis. It is suggested that research studies be carried out that may help in responding to this challenge.

## Figures and Tables

**Table 1:** Results of changes that occurred in the variables of the family structural dimension and their direction.

	What Changed from 0 = Nothing Changed to 7 = Everything Changed			If Change Is Greater Than or Equal to 4		
	n	Mean	SD	Frequency (nº)	Percentage (%)	
					Increased	Decreased
**Family income**	234	2.51	1.70	73	32.9	67.1
**Intensity of contact with extended family**	187	5.29	1.92	187	3.7	96.3
**Support to the household from extended family members**	234	3.71	2.14	Social Companionship (118)	22	78
				Emotional support (108)	35.2	64.8
				Material help and services (102)	30.4	69.6
**Interaction with coworkers**	234	4.71	1.91	163	11.7	88.3
**Interaction with friends**	234	5.75	1.56	201	1	99
**Interaction with health institutions**	234	3.97	2.06	127	7.1	92.9
**Interaction with educational institutions**	234	4.53	2.22	153	10.5	89.5
**Interaction with religious institutions**	234	3.71	2.48	114	3.5	96.5
**Interaction with community groups**	234	4.18	2.45	130	1.5	98.5
**Frequency of leisure activities**	234	5.74	1.58	200	2	98
**Frequency of cultural activities**	234	6.04	1.54	208	1	99
**Use of heating/air conditioning**	234	3.77	2.24	125	96	4
**Gas consumption**	234	3.28	2.27	107	92.5	7.5
**Water consumption**	234	4.16	2.09	147	98.6	1.6

**Table 2:** Results of correlations between employment changes and the structural dimension variables of the MDAIF.

What Changed in the Employment Status	n = 115	Employment Status	Family Income	Intensity of Contact with Extended Family	Support from Extended Family	Interaction with Coworkers	Interaction with Friends	Interaction with Health Institutions
**Unemployment**	6	102.17	98.25	52.83	44.67	56.67	74.5	64.67
**Retirement**	3	42.17	81.83	70.17	54.50	100.50	55.00	82.00
**Starting a work contract**	4	70.63	79.75	47.25	52.13	56.00	25.75	97.75
**Starting a service contract**	4	58.00	47.5	72.13	18.00	64.38	52.75	30.75
**Furlough**	12	61.88	71.54	55.42	65.54	49.21	50.25	55.17
**Telwork**	80	53.84	49.56	56.67	58.46	57.24	58.68	57.61
**Other**	6	61.00	83.83	77.75	82.42	62.83	74.50	41.83
**Kruskal–Wallis test (*p* value)**		13.913 (0.031)	24.032 (0.001)	4.305 (0.636)	11.037 (0.087)	6.275 (0.393)	8.174 (0.226)	11.961 (0.063)
**What changed in the employment status**	n = 115	Interaction with educational institutions	Interaction with community groups	Frequency of leisure activities	Frequency of cultural activities	Use of heating/air conditioning	Gas consumptions	Water consumption
**Unemployment**	6	56.00	36.42	55.17	54.67	58.08	43.08	57.25
**Retirement**	3	81.50	76.67	39.33	35.67	24.00	21.50	25.00
**Starting a work contract**	4	44.00	76.25	42.13	31.75	69.13	49.00	65.50
**Starting a service contract**	4	37.63	49.63	47.88	62.00	24.75	21.50	41.38
**Furlough**	12	45.13	42.08	44.42	35.83	43.75	67.33	59.13
**Telework**	80	60.03	61.01	61.59	63.03	61.09	62.11	59.27
**Other**	6	69.92	55.42	66.75	64.67	77.00	48.08	62.17
**Kruskal–Wallis test (*p* value)**	-	6.800 (0.340)	8.972 (0.175)	6.249 (0.396)	14.217 (0.027)	12.824 (0.046)	13.341 (0.038)	4.501 (0.609)

## Data Availability

The data presented in this study are available upon request from the corresponding author.
